# Typhoid Fever as a Cause of Liver Failure in the United States: A Case Report

**DOI:** 10.1155/crgm/3087201

**Published:** 2025-01-13

**Authors:** Syed Mujtaba Baqir, Neha Sharma, Aruge Lutaf, Monica Ghitan, Yu Shia Lin

**Affiliations:** ^1^Department of Medicine, Maimonides Medical Center, Brooklyn, New York 11219, USA; ^2^Department of Gastroenterology, Maimonides Medical Center, Brooklyn, New York 11219, USA; ^3^Department of Cardiology, Maimonides Medical Center, Brooklyn, New York 11219, USA; ^4^Department of Infectious Diseases, Maimonides Medical Center, Brooklyn, New York 11219, USA

**Keywords:** acute liver failure, extensively drug-resistant, multidrug-resistant, typhoid fever

## Abstract

**Background:** Typhoid fever is a multisystemic illness caused by *Salmonella typhi* and *Salmonella paratyphi*, transmitted fecal orally through contaminated water and food. It is a rare diagnosis in the US, with most cases reported in returning travelers. Hepatitis and cholestasis are rare sequelae of *salmonella* infection. However, acute liver failure (ALF) is exceptionally uncommon. We report a case of typhoid fever in a returning traveler to the US progressing to ALF.

**Case Presentation:** A 48-year-old man presented with high-grade fever, abdominal pain, vomiting, acholic stools, dark urine, and yellowish discoloration of skin and sclera for one week. He was immune to hepatitis A and B, with no recent change in medications. He had no history of alcohol consumption. On presentation, the patient was tachycardic but well perfused with diffuse abdominal tenderness. Laboratory results showed leukocytosis, elevated creatinine, mixed hepatocellular and cholestatic pattern of raised liver enzymes, elevated ammonia levels, and negative hemolytic parameters. Viral, autoimmune, and metabolic causes of hepatitis were negative. Ultrasound of the abdomen revealed a normal biliary system and a computerized tomography (CT) scan of the abdomen showed multiple liver cysts, mesenteric and porta-hepatis lymphadenopathy, and mild thickening of the terminal ileum. Intravenous (IV) ceftriaxone and metronidazole were initiated. Blood cultures grew *S*. *typhi*. The patient clinically deteriorated and developed altered mental status, respiratory distress, and an up-trending Model for End-Stage Liver Disease (MELD) score and was upgraded to the intensive care unit. IV meropenem was initiated, resulting in clinical recovery and negative repeat blood cultures. The patient completed 2 weeks of meropenem and was discharged.

**Conclusion:** Typhoid fever can cause life-threatening liver failure which is rare. Clinicians should be aware of this due to the rapid progression and life-threatening clinical course, as well as the rise of multidrug-resistant and extensively drug-resistant typhoid causing delays in starting the right antibiotic.

## 1. Introduction

Typhoid fever is an infectious multisystemic illness caused by *Salmonella typhi* and *Salmonella paratyphi*, transmitted fecal orally through contaminated water and undercooked food. It is an important health concern, especially in tropical and developing countries [1, 2]. It is a rare diagnosis in the United States, with most cases reported in returning travelers [2–4]. Hepatitis and cholestasis are a rare sequelae of *salmonella* infection reported in the literature [1–3, 5]. However, acute liver failure (ALF) secondary to typhoid fever is exceptionally uncommon. We report a case of typhoid fever in a returning traveler to the US progressing ALF.

## 2. Case

**Figure 1:**
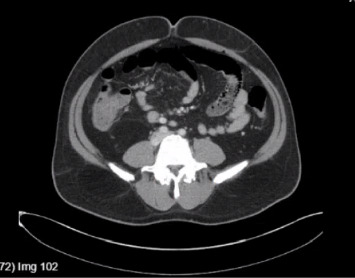
Mesenteric lymphadenopathy.

**Figure 2:**
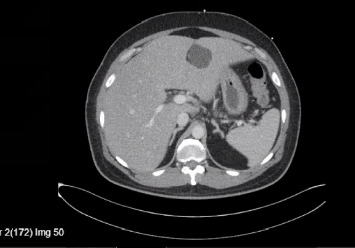
Porta-hepatis lymphadenopathy.

**Figure 3:**
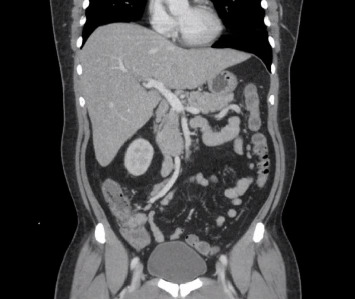
Terminal ileitis.

A 48-year-old man presented with high-grade fever with chills, abdominal pain, nausea, vomiting, acholic stools, dark urine, and yellowish discoloration of skin and sclera for one week. His past medical history was significant for diabetes and hypertension. He was immune to hepatitis A and B, with no history of bleeding diathesis nor recent use of new medications. He is a nonsmoker with no history of alcohol consumption. He returned from Pakistan 6 days prior to this admission. On presentation, the patient was ill-appearing, normotensive, and tachycardic (no relative bradycardia) but well perfused with diffuse abdominal tenderness and icteric sclera. Laboratory results showed leukocytosis and elevated creatinine. Liver function tests showed a mixed hepatocellular and cholestatic picture, elevated ammonia levels, and negative hemolytic laboratory parameters (Table 1). Workups for viral hepatitis, autoimmune hepatitis, and metabolic causes of hepatitis were negative, and serum salicylate levels were within normal range. Ultrasound of the abdomen revealed normal bile duct and gallbladder, and the absence of ascites. Doppler ultrasound ruled out thrombus. The echocardiogram revealed an ejection fraction of 61%–65%. A computerized tomography (CT) scan of the abdomen showed multiple liver cysts, mesenteric (Figure 1) and porta-hepatis lymphadenopathy (Figure 2), mild thickening of the terminal ileum (Figure 3) and proximal ascending colon. Magnetic resonance cholangiopancreatography (MRCP) showed severe hepatic steatosis and multiple scattered liver cysts. Empirically ceftriaxone and metronidazole were initiated after obtaining blood cultures. Blood cultures grew *Salmonella typhi*. Intravenous (IV) ceftriaxone was continued. The patient clinically deteriorated on the above treatment and developed altered mental status, respiratory distress, and showed signs of ALF with an up-trending Model for End-Stage Liver Disease (MELD) score. He was upgraded to the intensive care unit on Day 3 of admission, requiring on and off high-flow nasal cannula. His blood pressure remained within normal limits. Initially, IV ertapenem was started; however, the patient did not clinically improve and was not clearing the bacteria in the blood cultures, so the antibiotic was switched to IV meropenem on Day 5 of hospitalization due to its frequency of dosing and ability to achieve a comparatively better time to minimal inhibitory concentration (MIC), resulting in clinical recovery (creatinine improved, with hepatorenal syndrome ruled out) and clearance of infection on repeat blood cultures. The first blood cultures drawn grew *Salmonella typhi* resistant to ceftriaxone and ciprofloxacin. It was sensitive to azithromycin. No results were available for carbapenems. The patient completed 2 weeks of IV meropenem in-patient and was discharged after 22 days of hospital stay, stable on discharge, off any antibiotics, with negative blood cultures. One week after discharge, the patient was admitted due to back pain; however, his liver enzymes continued to remain within normal limits.

## 3. Discussion

ALF is characterized by severe acute liver injury, altered mental status, and impaired synthetic liver function (represented by INR > 1.5) in a person without known liver disease [6]. Viral hepatitis (hepatitis B, C, and E) and drug-induced liver injury (DILI) constitute the two most common etiologies of ALF overall, the former being more common in the developing world while the latter occurring more commonly in the developed world [6].

Approximately 400 cases of typhoid fever were reported in the United States between 2016 and 2018 with approximately 85% attributed to international travel [1]. This number declined in 2020 owing to the coronavirus pandemic and travel restrictions. 82% of these patients were hospitalized; however, no deaths were reported [1]. Nearly 75% of these patients had traveled from Pakistan or India to the United States [1].

Gastrointestinal complications of typhoid fever such as perforation of terminal ilium, ulceration, constipation, and obstruction have been commonly reported [1]. Intra-abdominal infections such as hepatic and splenic abscesses, pneumonia, lung abscesses, joint and bone infections, and neurological manifestations are other complications reported [1]. This case shows the potential of typhoid fever to cause life-threatening liver failure. As described in the case above, our patient had the characteristic liver injury, derangement of the synthetic liver function represented by the INR values, and altered mental status with other causes of chronic liver disease ruled out.

The production of typhoid toxin causes DNA damage within the hepatocytes, inducing cell apoptosis and necrosis [7]. Host immune response, which includes the proliferation of phagocytes and the formation of typhoid nodules, further contributes to hepatocyte damage [7]. These nodules represent marked hyperplasia of reticuloendothelial cells and can clog liver capillaries contributing to ischemic necrosis of liver cells. The subsequent coagulopathy can cause microvascular thrombosis causing further ischemia [7].

More profound jaundice with typhoid fever is rare, and its presence in prior cases reported has been associated with coinfection with hepatitis A and hepatitis E, leading to fulminant hepatitis [8]. On the contrary, our patient had extremely elevated bilirubin and transaminases with other causes of hepatitis and ALF ruled out. The prior case of enteric fever causing ALF was due to the growth of *Salmonella paratyphi* where the patient required ICU-level support [9]. However, our patient grew *Salmonella typhi*. The first-line treatment or empiric therapy is fluoroquinolones or third-generation cephalosporins. However, we now have multidrug-resistant (MDR) and extensively drug-resistant (XDR) strains of *Salmonella* [4] susceptible to only azithromycin and carbapenems. This strain was first reported in Pakistan in 2016 and has since been imported to the USA [4]. In Pakistan, 70% of the isolates were XDR in 2020 resistant to chloramphenicol, ampicillin, cotrimoxazole, ciprofloxacin, and third-generation cephalosporins [1]. Furthermore, no epidemiological studies have been conducted to detect the extent of the spread of this strain to other countries; however, in 2019, nearly 16 countries had imported this strain [1]. Between November 2019 and October 2020, 19 cases of XDR in the USA were identified without a travel history [1]. Our patient presented with a similar travel history, with symptoms commencing in Pakistan and progressing upon arrival to the United States. Apart from supportive treatment and consultation for a liver transplant, an essential principle in the management of ALF is to control the factor that incited the clinical event. In our case, the antibiotic was switched from ceftriaxone to meropenem, resulting in clinical recovery.

We conclude that our patient developed ALF secondary to typhoid fever, which is extremely rare; however, clinicians should develop a thought process around this due to the rapid progression and life-threatening clinical course as well as the rise of MDR and XDR typhoid causing delays in starting the right antibiotic.

## Figures and Tables

**Table 1:** Pertinent laboratory investigations.

	Day 1	Day 6	Day 7	Day 11	Day 15	Day 20 (discharge)	Day 25 (outpatient follow-up)
Total bilirubin (mg/dL) (range 0.2–1.4)	28.1	54.2	58.7	41.7	19.7	8.8	4.6
Direct bilirubin (mg/dL) (range 0–0.2)	20.9	43.0	48.1	25.9	9.2	4.0	2.1
AST (IU/L) (range 10–33)	94	69	58	63	80	70	38
ALT (IU/L) (range 6–47)	114	45	43	33	70	105	56
Alkaline phosphatase (IU/L) (range 36–112)	367	449	406	218	292	453	219
INR (range 0.8–1.2)	1.4	2.2	2.0	1.2	1.1	1.1	1.0

## Data Availability

All data generated or analyzed during this study are included in this published article.
